# Optimizing Class Imbalance in Facial Expression Recognition Using Dynamic Intra-Class Clustering

**DOI:** 10.3390/biomimetics10050296

**Published:** 2025-05-08

**Authors:** Qingdu Li, Keting Fu, Jian Liu, Yishan Li, Qinze Ren, Kang Xu, Junxiu Fu, Na Liu, Ye Yuan

**Affiliations:** 1 Institute of Machine Intelligence, University of Shanghai for Science and Technology, Shanghai 200093, China; liqd@usst.edu.cn (Q.L.);; 2 School of Optoelectronic Information and Computer Engineering, University of Shanghai for Science and Technology, Shanghai 200093, China

**Keywords:** facial expression recognition, class imbalance, dynamic intra-class clustering algorithm, deep learning

## Abstract

While deep neural networks demonstrate robust performance in visual tasks, the long-tail distribution of real-world data leads to significant recognition accuracy degradation in critical scenarios such as medical human–robot affective interaction, particularly the misidentification of low-frequency negative emotions (e.g., fear and disgust) that may trigger psychological resistance in patients. Here, we propose a method based on dynamic intra-class clustering (DICC) to optimize the class imbalance problem in facial expression recognition tasks. The DICC method dynamically adjusts the distribution of majority classes by clustering them into subclasses and generating pseudo-labels, which helps the model learn more discriminative features and improve classification accuracy. By comparing with existing methods, we demonstrate that the DICC method can help the model achieve superior performance across various facial expression datasets. In this study, we conducted an in-depth evaluation of the DICC method against baseline methods using the FER2013, MMAFEDB, and Emotion-Domestic datasets, achieving improvements in classification accuracy of 1.73%, 1.97%, and 5.48%, respectively. This indicates that the DICC method can effectively enhance classification precision, especially in the recognition of minority class samples. This approach provides a novel perspective for addressing the class imbalance challenge in facial expression recognition and offers a reference for future research and applications in related fields.

## 1. Introduction

Deep neural networks (DNNs) [1] have demonstrated remarkable advancements in vision tasks such as image classification [2], object detection [3,4], and semantic segmentation [5,6], with their success heavily reliant on large-scale, high-quality datasets [7,8]. However, real-world datasets commonly exhibit long-tailed distribution issues [9], where a small number of dominant categories constitute the majority of samples, while most categories suffer from severe scarcity. This imbalanced distribution poses significant challenges to DNN training [10], resulting in models that achieve superior performance on data-rich classes but exhibit substantial degradation in recognizing underrepresented categories (e.g., critical negative emotions like fear and disgust in medical human–robot affective interactions)—a phenomenon termed class imbalance [11]. In the context of clinical psychological interventions, facial expression recognition technology is widely applied in the auxiliary diagnosis and emotional monitoring of mental disorders such as depression, anxiety, and schizophrenia [12]. However, the class imbalance in the existing seven-class facial expression datasets significantly degrades model performance, particularly by reducing the recognition accuracy of low-frequency negative emotions and triggering misdiagnosis risks. This technical flaw not only restricts medical robots’ ability to detect patients’ subtle facial expressions but also weakens their emotional computing efficacy, thus limiting the clinical applicability of mental health intervention systems.

Due to the natural skew in emotion distribution and the influence of culture differences, real-world facial expression datasets (i.e., FER2013 [13,14] and AffectNet [15,16]) usually exhibit significant imbalance. Such class imbalance issues significantly hinder the generalization capability of facial expression recognition models and their precision in identifying rare expressions. To address this problem, early research focused on resampling and reweighting strategies, aiming to balance the influence of different categories by adjusting the distribution of training data. These methods optimize the training process by evaluating the importance of samples and incorporating techniques such as log-regularization to alleviate distribution discrepancies between training and test data, thereby enhancing the recognition performance of tail classes. However, an excessive emphasis on tail classes could potentially undermine the model’s ability to learn the features of head classes, leading to a decrease in their recognition accuracy.

Recent studies have indicated that the mismatch between representation and classifier learning plays a pivotal role in long-tail recognition [17,18]. To address this issue, several optimization methods have been proposed by researchers. Kang et al. [19] introduced a two-stage framework that decouples representation learning and classifier training. In the first stage, feature representations are learned using instance-balanced sampling; in the second stage, after the feature representations are fixed, the classifier is retrained using class-balanced sampling. This approach achieved significant improvements on long-tailed datasets such as ImageNet-LT and iNaturalist. Zhang et al. [20] proposed a distribution correction framework that effectively enhances the performance of long-tailed classification tasks, particularly on tail classes, by adjusting the distribution discrepancy between feature extraction and the classifier. Fan et al. [21] presented a joint learning framework, CoSSL, which significantly improves the generalization performance on tail classes by jointly optimizing the representation learning module and the classifier module, addressing the limitations of conventional two-stage methods in pseudo-label updates. Zhang et al. [20] proposed a unified distribution alignment strategy, which adjusts classification scores through an adaptive calibration function and introduces a generalization reweighting method with class prior balancing to enhance the performance of long-tailed visual recognition. Liu et al. [22] proposed learning shared latent features and performing semantic data augmentation to improve the representation ability of tail classes and mitigate the mismatch problem between the representation and classifier.

Inspired by the above works, our study builds upon the two-stage training strategy and proposes a dynamic intra-class clustering (DICC) algorithm to address class imbalance in facial expression recognition datasets. This method aims to enhance the classification performance of facial expression recognition models on imbalanced datasets. It does this by adjusting the distribution of long-tailed facial expression data in the feature space through dynamic clustering. This process boosts the model’s ability to learn from tail classes. The specific objectives are: (1) To present a novel DICC algorithm. This algorithm optimizes the model’s feature learning and classification performance by dividing the majority class into subclasses and assigning pseudo-labels. (2) To verify the effectiveness of the DICC algorithm on different facial expression datasets, especially in terms of its ability to recognize minority class samples.

Unlike conventional clustering methods [23,24] that employ static subclass partitioning and predefined cluster numbers, our DICC method introduces two pivotal innovations: (1) A dynamic centroid updating mechanism via the exponential moving average (EMA) method, enabling real-time synchronization between cluster centroids and evolving feature representations during training. This contrasts sharply with traditional k-means-based clustering that freezes centroids after initialization. (2) Adaptive interval partitioning that dynamically adjusts intra-class cluster boundaries based on real-time feature dispersion, reducing sensitivity to initial hyperparameter settings compared to static clustering methods. This dual innovation addresses the critical limitation of prior cluster-based methods—their inability to adapt to dynamically changing feature spaces in deep neural networks.

The main contributions are as follows: (1) We propose a dynamic intra-class clustering (DICC) method, which introduces a novel framework to address the class imbalance issue in facial expression datasets. (2) Our framework significantly improves performance by clustering the majority classes (i.e., head classes) into several subclasses, assigning unique pseudo-labels, and then mapping predictions back to the true labels. (3) Experiments demonstrate that our framework outperforms the state-of-the-art performance on long-tail expression datasets such as FER2013 [13,14], MMAFEDB [25,26], and Emotion-Domestic.

## 2. Related Works

Expression recognition aims to determine the emotional state of an individual by analyzing the shape, position, and dynamic changes of key facial components such as eyes, mouth, and eyebrows. This process is highly dependent on accurate and efficient facial feature extraction. To achieve effective feature extraction on unbalanced datasets, numerous sophisticated approaches have been proposed, and two solutions to the class imbalance problem have been gradually formed, namely, resampling [27] and reweighting [28].

### 2.1. Resampling

Resampling aims to balance the distribution of training data by either oversampling the tail classes or undersampling the head classes. Common resampling methods include oversampling [29,30] and undersampling [31,32]. However, although oversampling increases the number of samples in the minority classes (i.e., tail classes), it is completely dependent on the data enhancement method. The oversampled samples obtained by simple data enhancement methods such as rotation, shearing, and translation may be highly similar to the original data, and there is no essential performance improvement for model training, which can easily lead to overfitting. At the same time, the model learning effect may not be good due to the low quality of samples generated by oversampling. The undersampling method usually selects and discards samples in the majority classes according to certain rules, so that the overall distribution of the dataset is balanced, but this method is usually prone to losing a lot of sample information that is useful for model learning, which ultimately affects the learning performance of the model.

### 2.2. Reweighting

The reweighting method assigns different weights to categories to reduce the dominant role of the majority classes in model training and make the model pay more attention to the minority classes. For example, Lertnattee et al. [33] proposed the inverse class frequency (ICF) weighting method, which assigns weights inversely proportional to class sample sizes to reduce the dominance of majority classes. Cui et al. [34] introduced class-balanced loss, refining this approach by further scaling weights to improve minority class recognition. Lin et al. [35] developed focal loss, dynamically adjusting weights based on predicted probabilities to prioritize harder-to-classify samples. In addition, Cao et al. [36] proposed label distribution aware margin (LDAM), which calculates margins inversely proportional to the fourth root of class sample sizes based on label distribution, effectively reducing generalization error for minority classes. The common goal of these methods is to alleviate the excessive influence of the majority classes on the loss function and ensure that the model can learn from all categories in a balanced manner, thereby improving the overall classification performance.

### 2.3. Clustering Algorithms

The core idea of clustering is to automatically divide a dataset into several subsets (i.e., clusters) based on the intrinsic similarity measure between data points under an unsupervised learning framework, so that the similarity between sample features in the same cluster is maximized, while the similarity between different clusters is minimized. This process evaluates the similarity between features based on a certain distance metric (such as Euclidean distance, Manhattan distance, etc.), and completes classification by continuously iteratively optimizing and adjusting the cluster boundaries and the attribution of data points.

In recent years, clustering-based imbalanced classification tasks have received widespread attention. Wu et al. [37] proposed a method based on the nearest-neighbor strategy, employing locality information within clusters to balance the distribution of minority classes. However, this approach, while effective for local data distributions, may face limitations in addressing global feature structures, leading to suboptimal optimization of overall data characteristics. Guru et al. [38] adopted a subdivision strategy that further partitions each large category into finer subcategories, classifying them based on specific feature measurement methods. While this strategy focuses on dimensionality reduction in text data and offers insights for handling imbalance in high-dimensional datasets, its applicability remains constrained to specific scenarios. Harada et al. [39] proposed a semi-supervised domain adaptation framework for medical image classification, leveraging weakly-supervised clustering to obtain high-purity clusters for representation learning. While their approach enhances robustness to class imbalance, it may be sensitive to the quality of the clustering process and the availability of labeled data. Jiang et al. [40] introduced a semi-supervised hybrid resampling (SSHR) method that employs hierarchical clustering to capture data distribution for both oversampling and undersampling. By utilizing unlabeled data in minority clusters for oversampling, their method effectively addresses intra-class imbalance. However, the iterative cluster-splitting process may increase computational complexity and sensitivity to initial cluster configurations. Ju et al. [41] developed the C³GNN framework, integrating clustering into contrastive learning for class-imbalanced graph classification. By clustering graphs from each majority class into multiple subclasses and employing Mixup techniques, their method enhances semantic diversity and balances class distributions. Nonetheless, the approach’s effectiveness may be influenced by the choice of clustering algorithm and the complexity of graph structures.

Inspired by previous research, we propose embedding clustering methods into the training process of deep learning neural networks to optimize the class imbalance problem in facial expression recognition datasets. Specifically, features learned by the network are utilized for dynamic clustering to generate corresponding pseudo-labels, which are then fed back into the network to refine its training process. Ultimately, a label mapping network is employed to establish the association between pseudo-labels and their corresponding true labels, achieving a mapping from virtual to real labels. This approach enables more fine-grained classification of imbalanced facial expression data, effectively alleviating the issue of imbalanced expression classification. Compared to prior methods, this study introduces dynamic intra-class clustering to partition the majority classes into subclasses and integrates pseudo-labels with a label mapping module. This strategy dynamically adjusts the data distribution while optimizing feature learning and classification performance, making it more effective in handling the complexity of long-tailed data and addressing class imbalance challenges, thereby overcoming the limitations of previous approaches.

## 3. Method

Considering a training set, $D={\left\{\left(x_{i},y_{i}\right)\right\}}_{i=1}^{N}$, where *N* denotes the number of examples, $x_{i}$ denotes the *i*-th sample, and $y_{i}\in \left\{1,2,\ldots ,C\right\}$ denotes its corresponding label. The total number of training samples is $N=\sum_{c=1}^{C}N_{c}$, where *C* represents the number of categories and $N_{c}$ indicates the number of training samples belonging to the *C*-th category. For each training sample $\left(x_{i},y_{i}\right)$, the feature extraction in our method can be described as follows:

(1) $$f_{i}=\varphi \left(\theta ,x_{i}\right)$$

where θ represents the parameters of the network φ(·), and $f_{i}\in R^{M}$ denotes the *M*-dimensional feature. In the case of class imbalance, due to the limited number of samples in minority classes, it is difficult to identify key features that significantly distinguish them from majority classes in terms of feature quality. This results in indistinct features between minority and majority class samples, along with an imbalanced distribution, which is detrimental to subsequent classification tasks. Therefore, the objective of facial expression recognition tasks with a long-tailed distribution is to learn a deep neural network parameterized by θ. This network aims to achieve good recognition performance on a balanced test set.

After obtaining the image features, a linear classifier is utilized to establish a mapping from features to prediction probabilities for each class, with the class having the maximum probability value being selected as the predicted expression $y^{\prime }$ of the sample. The prediction probability *p* for each class is calculated as follows:

(2) $$p\left(x_{i}\right)=W\left(f_{i}\right)+b$$

where $W\in R^{M\times C}$ denotes the linear parameters of the classifier, and $b\in R^{C}$ represents the bias term. The weights *W* of the classifier are iteratively optimized and updated using the gradient descent algorithm. Due to the dominance of the majority class samples, their gradient contributions significantly outweigh those of minority class samples during backpropagation. Consequently, when the majority classes reach convergence saturation, the minority classes may still not have fully converged and require further training; alternatively, when the minority classes reach convergence saturation, the majority classes may already be overfitted.

**Figure 1:**
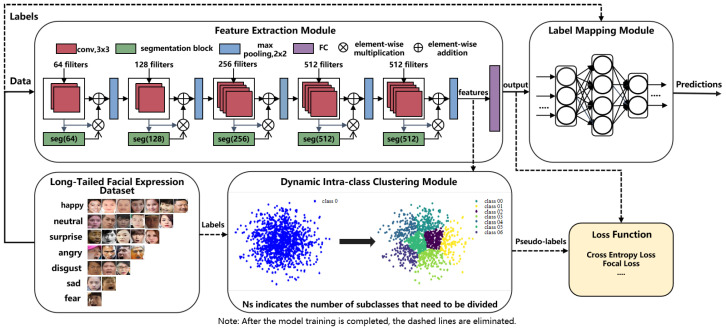
Overall framework diagram.

Here, we propose a facial expression recognition framework that comprises a feature extraction module, a dynamic intra-class clustering module, and a label mapping module, as shown in Figure 1. It splits the majority of sample categories into subclasses to learn a balanced feature extractor, and optimizes the weights of classifiers to accommodate these subclasses. Additionally, a two-stage training strategy is employed to update the weights of the entire algorithmic framework. During the training process, the feature extraction module utilizes the VGG19 backbone network to extract features from the input images. Subsequently, the dynamic intra-class clustering module assigns pseudo-labels to the features based on their intra-class distances. Finally, the label mapping module maps these pseudo-labels to the true labels. During the inference and testing phases, the dynamic intra-class clustering module is removed.

### 3.1. Dynamic Intra-Class Clustering Module

The dynamic intra-class clustering module operates within the image representation learning phase, dividing the sample features into a specified number of subclasses based on their similarity, thereby ensuring that classes with imbalanced samples maintain a balanced distribution in the feature space. As head classes are typically distributed in the dense regions of the feature space, while tail classes are concentrated in the sparse regions, the DICC module further refines the classification of sample features into subclasses, thereby improving the ability of the model to learn features in more dimensions:

1. The head classes are associated with high-density regions in the feature space, which can be interpreted through the clustering of subcategories. Initially, many subcategories are distributed across many small classes, which reduces the overall feature density. This can be formulated as follows:
  (3) $$D_{\mathrm{head}}=\overset{{N_{s}}^{k}}{\underset{k=1}{⋃}}D_{\mathrm{head}}^{k}\;\mathrm{and}\;∥D_{\mathrm{head}}^{k}∥\;\approx \;∥D_{\mathrm{tail}}∥$$
  where $D_{\mathrm{head}}^{k}$ represents the *k*-th subclass of the head class, and ${N_{s}}^{k}$ is the number of subcategories. $D_{\mathrm{head}}$ and $D_{\mathrm{tail}}$ represent the complete sample sets of the original head and tail classes.
2. In the training process, the DICC module increases the complexity of the classification task, thereby compelling the classifier to distinguish among more fine-grained subclasses. This process helps to balance the gradient contributions of each subclass.
3. The DICC module enables the feature extraction network to learn finer-grained local features. From an information-theoretic perspective, the introduction of subclass pseudo-labels increases the conditional entropy H(Y|X), thereby necessitating the maximization of the mutual information I(X;Y) between the feature representation $f_{\theta }\left(x\right)$ and the label *Y*. This mechanism drives the network to capture subtle differences in tail-class samples, such as local muscle movement variations between “fear” and “sadness” facial expressions.
  where the mutual information is defined as follows:
  (4) I(X;Y)=H(Y)−H(Y∣X)
  The conditional entropy H(Y|X) characterizes the uncertainty remaining in *Y* after observing *X*. The maximization of I(X;Y) enforces the feature representation to preserve discriminative information for fine-grained classification.

Specifically, during the training, this module receives the sample features extracted by a convolutional neural network as input data. For the features of each category within each batch of data, the module performs the following iterative process: (1) It calculates the center of the sample features for each category. (2) It determines the maximum distance from each sample feature to the center of its respective category’s features. This maximum distance value is then divided into several sub-intervals. (3) Based on the distance of each sample feature to the center of its category’s features (sorted in ascending or descending order), the module determines the sub-interval in which the feature falls and assigns a corresponding pseudo-label to each sample accordingly. These pseudo-labels are used for subsequent loss computation and model optimization. The process can be described as follows:

(5) $$d_{t}=\frac{\max|F_{i}-\mu_{t}|}{N}$$

(6) $$\mathrm{if}\left|F_{i}-\mu_{t}\right|\in \left(nd_{t},\left(n+1\right)d_{t}\right),\;n\in \left[0,1,\ldots ,N-1\right],\;F_{i}\in P_{n}$$

where $\mu_{t}$ refers to the sample center of a particular category in the *t*-th batch during training, $F_{i}$ denotes the *i*-th sample within that category, $P_{n}$ represents the feature sample set of the *n*-th subclass, and *N* indicates the number of subclasses divided within this category. That is, the maximum distance between the features and the feature center is uniformly divided into *N* intervals, each with a length of $d_{t}$.

To more effectively compute the sample feature center (i.e., the average position of features) for each category, the module employs the exponential moving average (EMA) method. It enables smooth updates of the sample feature center, gradually approximating it to the true center of the corresponding category within the entire dataset. When using a mini-batch training strategy, it can reduce computational costs and enhance the accuracy of the sample feature center. The calculation of the sample feature center is presented as follows:

(7) $$\mu_{t}=\left\{\begin{array}{cc} y_{t} & t=1\\ \alpha y_{t}+\left(1-\alpha \right)\mu_{t-1} & t>1 \end{array}\right.$$

where α∈(0,1) denotes the decay rate of the weight. $y_{t}$ denotes the average of the features for a particular category in the *t*-th batch. $\mu_{t}$ indicates the exponential moving average (EMA) of the features for a specific category in the *t*-th batch, which serves as the sample feature center. By introducing a decay factor α=0.99, a smooth weighted average of past sample feature centers is achieved, thereby making it closer to the overall sample center of that category within the dataset.

The number of subclasses Ns is crucial for balancing data distribution and enhancing classification accuracy. Based on the distribution characteristics of the dataset, we use the sample size of the tail category (i.e., the category with the smallest number of samples) as the basic unit for a single subclass and, accordingly, divide the head categories evenly to derive the number of subclasses for each category. The detailed experimental results are presented in Section 4.4. Theoretically, subdividing the majority categories in a highly imbalanced dataset into more subclasses can help achieve a balanced data distribution. However, through numerous experimental validations, we have found that more subclasses do not necessarily lead to better results. As the number of subclasses increases, the intra-class distance of samples tends to decrease, and an excessive number of subclasses can result in a decline in classification accuracy. Therefore, the approach to subclass division must be comprehensively considered in conjunction with the specific circumstances of the dataset to ensure both balanced data distribution and high classification accuracy. The calculation process for dynamic intra-class clustering is illustrated in Algorithm 1.
**Algorithm 1** Dynamic intra-class clustering (DICC)  1:**Input:** features extracted by convolutional neural networks, labels for the sample data, Ns is the number of sub-categories (must be divisible by the number of head classes M).  2:**Output:** newlabels are the pseudo-labels.  3:Initialize the feature center of the sample $\mu_{0}=0$  4:**for** t=1 **to** batches **do**  5:      **for** i=0 **to** classes **do**  6:            $loc\leftarrow \mathrm{where}(labels_{t}=i)$  7:            $y_{t}\leftarrow \mathrm{mean}\left(features_{t}\right)$  8:            $\mu_{t}\leftarrow \alpha y_{t}+\left(1-\alpha \right)\mu_{t-1}$  9:            $distance\leftarrow \;∥features_{t}-\mu_{t}∥$10:            index←argsort(distance)11:            $s\leftarrow \sum_{k=0}^{i}Ns\left[k\right]$12:            newlabels[loc]←index+s13:            i←i+114:      **end for**15:      t←t+116:**end for**

### 3.2. Label Mapping Module

To achieve an effective mapping from the pseudo-label space to the true-label space, we introduce a label mapping module after the feature extraction phase. This module, which employs a multi-layer fully connected network, aims to transform the label space accurately. During the training phase, since each class’s samples are further divided into multiple subclasses, the number of generated pseudo-labels will exceed the number of true labels. To address this, a three-layer fully connected network is designed, with the input dimension set to the total number of pseudo-labels and the output dimension equal to the total number of true labels. This design enables the network to learn the mapping from the expanded pseudo-label space to the original true-label space, ensuring the accuracy and effectiveness of the mapping process, thereby achieving precise alignment between pseudo-labels and true labels.

### 3.3. Training Strategy

**Figure 2:**
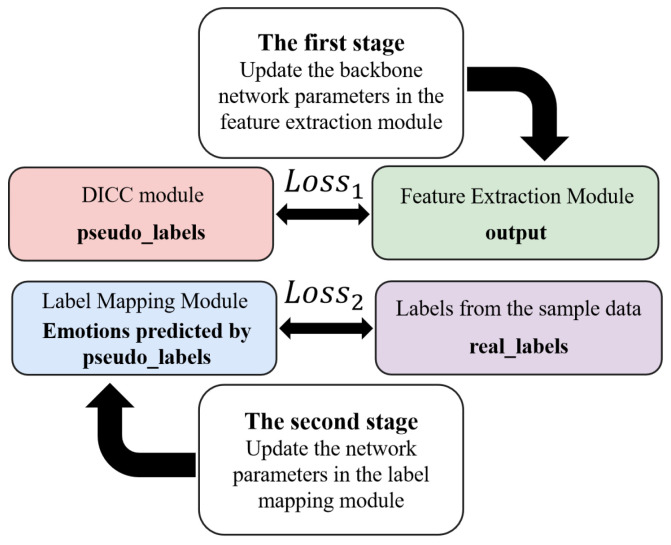
Two-phase training process.

A two-phase training strategy is designed to iteratively optimize the weight parameters of the framework. Specifically, the training process is divided into two phases. In the first phase, the pseudo-labels generated by the dynamic intra-class clustering module are used as the basis for calculating the loss of the backbone network. The pseudo-labels are compared with the benchmark pseudo-labels to compute the backbone network loss ($Loss_{1}$), based on which the weight parameters of the feature extraction module are updated, aiming to extract accurate and discriminative feature representations. In the second phase, the true labels of the samples are used as the ground truth, and the labels predicted by the label mapping module are compared with the true labels to compute the mapping network loss ($Loss_{2}$), based on which the weight parameters of the label mapping module are updated to achieve effective alignment between pseudo-labels and true labels. These two phases are executed alternately, ensuring that the model can make fine adjustments between the accuracy of feature extraction and the precision of label mapping. The schematic diagram of loss calculation for the two-phase training is shown in Figure 2.

## 4. Experiments and Analysis

### 4.1. Experimental Data

The FER2013 (Facial Expression Recognition 2013 Dataset) [13,14] is a widely utilized dataset for facial expression recognition. This dataset comprises 35,887 grayscale images, each of which has been preprocessed and resized to a dimension of 48×48 pixels. The images encompass seven fundamental facial expression categories, as follows: angry, disgust, fear, happy, sad, surprise, and neutral. The FER2013 dataset is partitioned into a training set consisting of 28,709 images, a validation set comprising 3589 images, and a test set containing 3589 images. Owing to its large scale and diversity, the FER2013 dataset serves as an ideal resource for training deep learning models.

The MMAFEDB dataset [25,26] is a comprehensive facial expression recognition dataset that encompasses multiple subsets. Primarily composed of facial expression data from individuals of European and American descent, it comprises a training set with 92,968 images, a validation set with 17,375 images, and a test set with 17,356 images, totaling over 127,000 images. The dataset includes the same seven fundamental expressions as its counterparts. Characterized by its large sample size, the MMAFEDB is well-suited for the training and validation of facial expression recognition models.

The Emotion-Domestic dataset is a facial expression dataset tailored to Asian individuals. It comprises static images sourced from online videos and annotated with seven basic emotions. This dataset is segmented into a training set, which contains 49,601 images, and a test set, comprising 4000 images. Its distinctive feature lies in its specificity to Asian faces, rendering it highly applicable for research on facial expression recognition within Asian populations.

**Figure 3:**
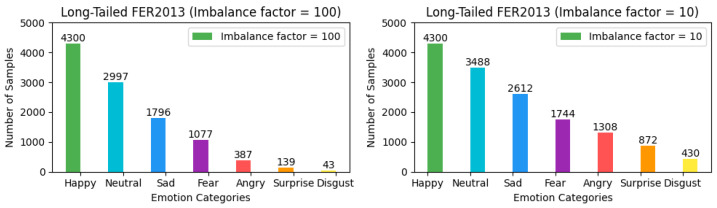
Illustration of class imbalance in the FER2013 dataset.

**Figure 4:**
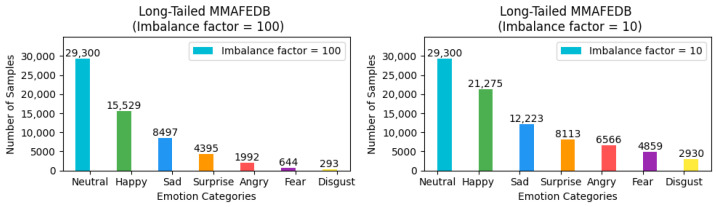
Illustration of class imbalance in the MMAFEDB dataset.

**Figure 5:**
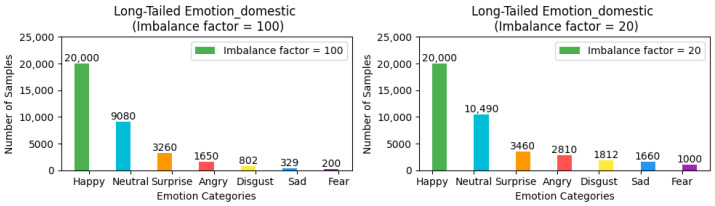
Illustration of class imbalance in the Emotion-Domestic dataset.

To ensure the rigor and feasibility of our experiments, we adjusted the distribution of the three aforementioned facial expression datasets—which inherently exhibit a long-tailed distribution—by introducing an imbalance factor $\beta =\frac{N_{\max}}{N_{\min}}$ to precisely control the degree of dataset imbalance. Here, $N_{\max}$ and $N_{\min}$ represent the number of samples with the highest and lowest frequency of occurrence in the training set, respectively. For the FER2013 and MMAFEDB datasets, we set two imbalance factors: 10 and 100, to simulate scenarios with varying degrees of data imbalance. For the Emotion-Domestic dataset, we selected 20 and 100 as the imbalance factors for adjustment. It is noteworthy that the distribution of the test set remains unchanged. This treatment simulates data imbalance conditions closer to real-world scenarios, thereby allowing for a more systematic verification of framework performance across datasets with different imbalance factors and a comprehensive assessment of model performance under varying degrees of imbalance. The preprocessed versions of all datasets, along with our imbalance adjustment scripts, are publicly available for research purposes. The dataset processing scripts and download links for the adjusted datasets have been archived in our GitHub Link https://github.com/fuketing0417/DICC-Facial-Expression-Recognition (default branch, accessed on 30 April 2025) This ensures full reproducibility of our experimental setup and facilitates comparisons with future studies. The adjusted long-tailed distribution is illustrated in Figure 3, Figure 4 and Figure 5.

### 4.2. Experimental Design

In this experimental setup, we employed the PyTorch 1.13.1 framework to construct a model using VGG19 [42] as the backbone network, with random weight initialization. The optimization process was carried out using the stochastic gradient descent (SGD) algorithm with a momentum of 0.9, over 200 epochs, and a learning rate of 0.0001. The experiments were conducted on devices equipped with NVIDIA RTX 3090 graphics cards (The cards are manufactured by NVIDIA Corporation, located in Santa Clara, CA, USA), using a batch size of 32.

#### 4.2.1. Configuration of Baseline Methods

To validate the effectiveness of the proposed method, this experiment compared it with state-of-the-art techniques commonly used to optimize classification on imbalanced datasets, as well as with their combinations, that is, (1) cross-entropy loss (CE Loss), which does not alter sample weights; (2) resampling, which adjusts the sampling frequency based on the inverse of the effective number of samples per class; and (3) reweighting, which adjusts the sample weights using the inverse of the effective number of samples per class as the weighting factor. The experiment utilized CE Loss in conjunction with three training methods (none, resample, and reweight) as different baseline training approaches.

#### 4.2.2. Setup of Evaluation Metrics

To comprehensively evaluate the performance of the proposed method, this study introduces a multi-dimensional set of evaluation metrics. First, Top-1 accuracy is employed as the primary indicator of overall performance. This metric is widely used in long-tail class-imbalanced learning scenarios and effectively reflects the model’s general classification ability. Additionally, to provide a more detailed view of the performance of the baseline method and the proposed DICC method across different classes, particularly in handling minority class samples, precision–recall curves are used to assess the model’s classification quality for each class. Finally, confusion matrices are utilized to visualize the model’s prediction results, providing a clear overview of the classification distribution and misclassifications across categories. This further aids in analyzing the model’s limitations and identifying potential directions for improvement.

### 4.3. Experimental Results

The experimental results indicate that across all long-tailed datasets with varying settings of the imbalance factor β and different combination strategies, the DICC method outperformed the baseline in terms of testing accuracy, particularly in the Emotion-Domestic dataset, where it demonstrated a significant improvement, with the maximum enhancement reaching approximately 5.48%. Specifically, in scenarios with more imbalanced data distributions, the accuracy increase of the DICC method was more pronounced compared to the baseline, suggesting its more prominent effectiveness in addressing more imbalanced distributions. Importantly, the DICC module introduces only a marginal latency increase of approximately 0.2 ms per batch during training and incurs no inference-time overhead. This is because the clustering operates solely in the feature space without modifying the backbone network’s inference pipeline.

Collectively, these results substantiate the efficacy of the DICC method in tackling class imbalance issues in facial expression recognition, exhibiting superior robustness and broader applicability over the baseline. The following three tables, namely Table 1, Table 2 and Table 3, present the testing accuracies of the research framework under different combination strategies for the long-tailed FER2013, MMAFEDB, and Emotion-Domestic datasets. All experiments were repeated with five different random seeds to ensure reliability. The test accuracies reported in Table 1, Table 2 and Table 3 represent the best results obtained from these five independent runs. The specific results from the five runs are detailed in Table A1, Table A2 and Table A3 in the Appendix A.

**Figure 6:**
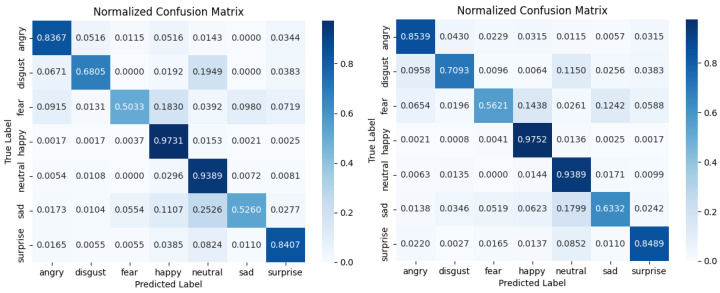
Confusion matrix between the DICC method and baseline on the long-tailed Emotion-Domestic dataset with imbalance factor β=20. Note: The (**left hand**) diagram presents the confusion matrix for the baseline model, while the (**right hand**) diagram exhibits the confusion matrix employing the DICC method.

**Figure 7:**
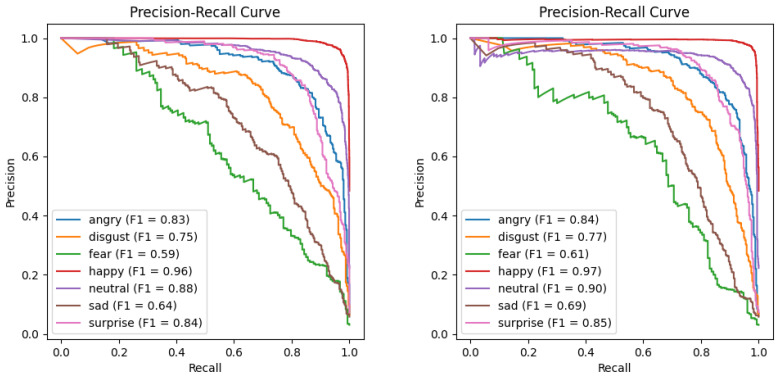
Precision–recall curves between the DICC method and baseline on the long-tailed Emotion-Domestic dataset with imbalance factor β=20. Note: The (**left hand**) diagram presents the precision–recall curves for the baseline model, while the (**right hand**) diagram exhibits the precision–recall curves with the DICC method.

To more vividly illustrate the superiority of the DICC method over the baseline on the long-tailed Emotion-Domestic dataset, this study conducted a comparative analysis using confusion matrices and precision–recall (PR) curves. Figure 6 demonstrates the performance discrepancies between the baseline and DICC methods across different categories. The confusion matrix indicates that the DICC method is more effective at reducing misclassifications in long-tailed distributions than the baseline method, with particularly significant improvements in the prediction accuracy for the categories of “sad” and “fear”. This suggests that the DICC method has a more pronounced advantage in alleviating the effects of data imbalance. Additionally, the PR curves in Figure 7 show that the DICC method achieves a markedly higher F1 score in most categories, with especially significant enhancements in recognizing challenging classes. This indicates that the intra-class clustering strategy of the DICC method effectively increases the precision and recall rates for minority classes, thereby enhancing the model’s generalization capabilities on long-tailed data. Collectively, these findings lead to the conclusion that the DICC method is more effective at improving model classification performance in tasks involving long-tailed emotional recognition compared to the baseline method.

### 4.4. Ablation Experiments Results

In preliminary experiments, this study observed that the performance of the model varied across categories as the number of subclasses, denoted as Ns, changed. This indicates that the number of subclasses plays a pivotal role in the effectiveness of the dynamic intra-class clustering method. However, the initial experiments were insufficient to conduct a comprehensive analysis of the impact of varying Ns. Consequently, this study further designed ablation experiments to systematically explore the effects of different configurations on model performance.

In the ablation study, while holding other parameters constant, the number of Ns was incrementally adjusted, and the classification accuracy on the test dataset was recorded for each configuration. Through these experiments, the aim was to determine the optimal number of subclasses for datasets with varying degrees of imbalance, thereby enhancing the model’s generalization capabilities and robustness. Partial results of these experiments are presented in Table 4 below.

The experimental results indicate that the classification accuracy of the model on the test dataset is not directly proportional to the number of subclasses. With the increase in the number of subclasses, the classification accuracy of certain combinations of baseline and DICC methods (such as CE + none) increased, while for certain combinations (such as CE + resample), the classification accuracy slightly decreased. Specifically, when the subclass number was set to [6,2,1,20,18,1,10], the model achieved the highest classification accuracy of 91.180%, suggesting that an appropriate number of subclasses can effectively enhance the model’s generalization and robustness against long-tailed datasets. However, when the subclass number was further increased to [8,4,1,40,24,1,12], the classification accuracy dropped to 91.260%, which may imply that overly granular subclass partitioning could lead to model overfitting, thereby affecting its performance on the test set. Therefore, it is evident that an excessive number of subclasses introduces unnecessary noise, reducing the discriminability between subclasses and impacting accuracy, while too few subclasses limit the richness of feature extraction. Consequently, considering the stability and performance of the model framework, the subclass partitioning strategies for different datasets are detailed in Table 5.

## 5. Discussion and Conclusions

This study introduces a dynamic intra-class clustering method aimed at enhancing the classification performance of long-tailed facial expression datasets. Experimental results on long-tailed datasets such as FER2013, MMAFEDB, and Emotion-Domestic demonstrate that this method excels in classification accuracy, particularly in the recognition capability of minority class samples, showcasing its strong potential in optimizing class imbalance issues in facial expression recognition tasks. Moreover, the results indicate that the framework proposed in this study can effectively integrate with existing rebalancing strategies, such as resampling and reweighting, further validating its robust applicability and versatility.

### 5.1. Limitations and Challenges

While the DICC method demonstrates promising results on benchmark datasets, several limitations and practical challenges warrant further discussion.

#### 5.1.1. Computational Complexity Analysis

The proposed dynamic clustering algorithm theoretically exhibits O(NKt) time complexity, where N denotes sample size, K represents subclasses, and t indicates iterations. This complexity pattern is empirically validated through our training time experiments (see Table 6):

- Sample size dependency (N): In the MMAFEDB dataset, our method exhibits 73.58% longer training time compared to the baseline under the CE+Resample (β=100) configuration (3.68 h vs. 2.12 h), which aligns with the linear scaling law of the O(N) term. This indicates that dynamic clustering introduces non-negligible computational overhead for large-scale facial datasets.
- Subclass interaction (K): The β=100 experiments on FER2013 demonstrate a 41.18% increase in the training time for our method (0.96 h vs. 0.68 h) under the CE + Reweight setting when subclass granularity intensifies (K↑). This empirically validates the practical impact of the O(K) complexity factor.

#### 5.1.2. Cross-Cultural Susceptibility to Feature Space Instability

Experimental results indicate that the DICC algorithm exhibits significant performance variations across datasets from different cultural backgrounds. Specifically, in the MMAFEDB dataset, which is constructed for Western populations (β=100, with the CE + Resample + DICC strategy), the classification accuracy of the DICC algorithm is improved by 1.97% compared to the baseline method. In stark contrast, in the Emotion-Domestic dataset, which is designed for Asian populations, the DICC algorithm, under the same strategy, achieves an accuracy improvement of 3.71% over the baseline method. This phenomenon may be attributed to the inherent differences in cross-cultural facial action coding systems (FACS) [43], particularly the systematic variations in facial expression intensity and regional muscle linkage mechanisms among different ethnic groups. These factors pose substantial challenges to the dynamic intra-class clustering mechanism of the DICC algorithm.

#### 5.1.3. Subclass Sensitivity

Our ablation studies (Table 4) reveal sensitivity to subclass granularity, that is, excessive partitioning introduces intra-class fragmentation, while insufficient subdivision limits feature discrimination. Current subclass allocation relies on manual configuration, constraining adaptability across datasets.

### 5.2. Future Directions

Therefore, future research should explore more efficient dynamic clustering algorithms to reduce computational overhead and optimize sub-class structures to decrease training costs. Additionally, subsequent studies could investigate the introduction of AU-aware [44] clustering constraints or establish culture-adaptive sub-class number determination mechanisms to address these challenges. It is also crucial to further develop automated optimization methods for determining the number of sub-classes and to investigate joint training strategies to enhance overall performance, thereby improving the model’s practical applicability and cross-cultural robustness.

### 5.3. Conclusions

In summary, the framework proposed in this study significantly improves the performance of models in long-tailed facial expression recognition tasks and provides new insights into optimizing class imbalance issues in facial expression recognition datasets. Future work will focus on optimizing dynamic clustering strategies and adaptive sub-class number adjustment mechanisms to further enhance the generalization capability and practical application effects of the method.

## Figures and Tables

**Table 1:** Test accuracy of the DICC method and baseline on the long-tail FER2013 dataset.

Dataset		Imbalanced FER2013		
β	**Loss**	**Rule**	**Baseline**	**Ours**
100	CE	None	52.522	**53.051**
		Resample	49.986	**50.850**
		Reweight	49.262	**50.293**
10	CE	None	60.769	**61.159**
		Resample	59.209	**60.936**
		Reweight	59.738	**60.379**

Note: Baseline: the model without our method; ours: the model with our method; β: imbalance factor; loss: the loss function of the model; rule: the training method of the model. There are several baselines due to the variety of loss functions and training methods. For each baseline, our method can be combined with it. The values in bold indicate that, under identical conditions, the accuracy of the model with our method is higher than the model without our method.

**Table 2:** Test accuracy of the DICC method and baseline on the long-tail MMAFEDB dataset.

Dataset		Imbalanced MMAFEDB		
β	**Loss**	**Rule**	**Baseline**	**Ours**
100	CE	None	55.157	**55.531**
		Resample	53.866	**54.926**
		Reweight	52.915	**53.676**
10	CE	None	56.263	**56.782**
		Resample	55.209	54.903
		Reweight	54.500	**54.823**

The meaning is the same as Table 1.

**Table 3:** Test accuracy of the DICC method and baseline on the long-tail Emotion-Domestic dataset.

Dataset		Imbalanced Emotion-Domestic		
β	**Loss**	**Rule**	**Baseline**	**Ours**
100	CE	None	84.660	**88.000**
		Resample	83.660	**86.760**
		Reweight	82.440	**86.960**
20	CE	None	88.920	**90.760**
		Resample	88.360	**90.980**
		Reweight	88.480	**90.600**

The meaning is the same as Table 1.

**Table 4:** Test accuracy for different numbers of subclasses in the long-tailed Emotion-Domestic dataset (β=20) under the DICC method.

Method	Subclass Number (Ns)			
	[2,1,1,5,3,1,2]	[2,1,1,10,8,1,4]	[6,2,1,20,18,1,10]	[8,4,1,40,24,1,12]
CE + None + DICC	90.800	90.760	91.180	91.260
CE + Resample + DICC	90.080	90.980	90.660	90.440
CE + Reweight + DICC	91.020	90.600	88.980	88.900

**Table 5:** Table of subclass numbers for different datasets under various imbalance factors.

Dataset	Imbalance Factor (β)	Subclass Number (Ns)
FER2013	100	[8,1,20,100,40,4,80]
	10	[4,1,6,10,8,2,8]
MMAFEDB	100	[6,1,2,60,100,30,20]
	10	[2,1,2,8,10,6,4]
Emotion-Domestic	100	[20,6,1,100,60,2,30]
	20	[2,1,1,10,8,1,4]

**Table 6:** Training time comparison across facial expression datasets.

Dataset	Configuration	Baseline (h)	Ours (h)	Increase (%)
FER2013	CE + Reweight (β=100)	0.68	0.96	+41.18
MMAFEDB	CE + Resample (β=100)	2.12	3.68	+73.58
Emotion-Domestic	CE + None (β=20)	1.37	1.75	+27.73

Note: Time increase percentage calculated as $\frac{\mathrm{Ours}-\mathrm{Baseline}}{\mathrm{Baseline}}\times 100\%$; due to space limitations, this presentation includes a selection of the comparative training times.

## Data Availability

The original contributions presented in this study are included in the article; further inquiries can be directed to the corresponding author.

## Appendix A

**Table A1:** Test accuracy of DICC with different imbalance factors across five random seeds on long-tailed FER2013.

Method	Baseline	Run1	Run2	Run3	Run4	Run5
CE + None (β = 100)	52.522	52.817	52.734	**53.051**	52.837	53.164
CE + Resample (β = 100)	49.986	50.324	**50.850**	50.412	50.742	50.728
CE + Reweight (β = 100)	49.262	49.865	50.127	50.166	49.845	**50.293**
CE + None (β = 10)	60.769	60.934	**61.159**	61.043	60.912	61.048
CE + Resample (β = 10)	59.209	59.825	**60.936**	60.274	60.125	60.815
CE + Reweight (β = 10)	59.738	60.158	60.271	60.012	60.004	**60.379**

Note: The bold-faced values correspond to the highest precision obtained in the testing process.

**Table A2:** Test accuracy of DICC with different imbalance factors across five random seeds on the long-tailed MMAFEDB dataset.

Method	Baseline	Run1	Run2	Run3	Run4	Run5
CE + None (β = 100)	55.157	55.412	55.337	55.468	**55.531**	55.284
CE + Resample (β = 100)	53.866	54.420	**54.926**	54.412	54.271	54.603
CE + Reweight (β = 100)	52.915	53.357	53.601	**53.676**	53.195	53.510
CE + None (β = 10)	56.263	**56.782**	56.518	56.634	56.429	56.725
CE + Resample (β = 10)	**55.209**	54.699	54.876	54.903	54.732	54.432
CE + Reweight (β = 10)	54.500	54.615	54.732	54.649	**54.823**	54.778

Note: The bold-faced values correspond to the highest precision obtained in the testing process.

**Table A3:** Test accuracy of DICC with different imbalance factors across five random seeds on the long-tailed Emotion-Domestic dataset.

Method	Baseline	Run1	Run2	Run3	Run4	Run5
CE + None (β = 100)	84.660	87.740	**88.000**	87.620	87.960	87.840
CE + Resample (β = 100)	83.660	**86.760**	86.680	86.520	86.660	86.720
CE + Reweight (β = 100)	82.440	86.840	86.760	86.800	**86.960**	86.740
CE + None (β = 20)	88.920	90.140	90.680	**90.760**	89.980	90.520
CE + Resample (β = 20)	88.360	90.820	**90.980**	90.740	90.560	90.680
CE + Reweight (β = 20)	88.480	90.460	90.400	90.540	90.480	**90.600**

Note: The bold-faced values correspond to the highest precision obtained in the testing process.

**Table A4:** Performance comparison on the long-tailed Emotion-Domestic dataset β=20.

Method	Precision		Recall		F1 Score	
	**Baseline**	**Ours**	**Baseline**	**Ours**	**Baseline**	**Ours**
CE + None	0.8151	0.8393	0.7630	0.7975	0.7851	0.8139
CE + Resample	0.8148	0.8473	0.7396	0.8037	0.7675	0.8229
CE + Reweight	0.8045	0.8367	0.7424	0.7918	0.7684	0.8120

**Table A5:** Performance comparison on the standard benchmark datasets.

Method	FER2013		MMAFEDB		Emotion-Domestic	
	**Baseline**	**Ours**	**Baseline**	**Ours**	**Baseline**	**Ours**
CE + None	65.478	**66.063**	60.351	**60.854**	91.040	**91.240**
CE + Resample	66.592	65.616	59.682	59.468	90.860	**91.440**
CE + Reweight	65.394	**65.918**	59.473	**59.921**	90.920	**91.680**

Note: The bold-faced values correspond to the highest precision obtained in the testing process.

**Table A6:** Test accuracy improvement comparison on the standard benchmark datasets.

Method	FER2013	MMAFEDB	Emotion-Domestic
CE + None	+0.89%	+0.83%	+0.22%
CE + Resample	−1.47%	−0.36%	+0.64%
CE + Reweight	+0.80%	+0.75%	+0.84%

**Table A7:** Test accuracy improvement comparison on the long-tailed datasets.

Method	FER2013		MMAFEDB		Emotion-Domestic	
	β=100	β=10	β=100	β=10	β=100	β=20
CE + None	+1.01%	+0.64%	+0.68%	+0.92%	+3.95%	+2.07%
CE + Resample	+1.73%	+2.92%	+1.97%	−0.56%	+3.71%	+2.97%
CE + Reweight	+2.09%	+1.07%	+1.44%	+0.59%	+5.48%	+2.40%
